# mGluR1 in cerebellar Purkinje cells is essential for the formation but not expression of associative eyeblink memory

**DOI:** 10.1038/s41598-019-43744-z

**Published:** 2019-05-14

**Authors:** Harumi Nakao, Yasushi Kishimoto, Kouichi Hashimoto, Kazuo Kitamura, Miwako Yamasaki, Kazuki Nakao, Masahiko Watanabe, Masanobu Kano, Yutaka Kirino, Atsu Aiba

**Affiliations:** 1 Division of Molecular Genetics, Department of Physiology and Cell Biology, Kobe University Graduate School of Medicine, Kobe, Hyogo 650-0017 Japan; 2 Laboratory of Animal Resources, Center for Disease Biology and Integrative Medicine, Graduate School of Medicine, The University of Tokyo, Bunkyo-ku, Tokyo 113-0033 Japan; 3 Laboratory of Neurobiophysics, Kagawa School of Pharmaceutical Sciences, Tokushima Bunri University, Sanuki, Kagawa 769-2193 Japan; 4 Department of Neurophysiology, Graduate School of Medicine, The University of Tokyo, Bunkyo-ku, Tokyo 113-0033 Japan; 5 Department of Neurophysiology, Graduate School of Biomedical & Health Sciences, Hiroshima University, Minami-ku, Hiroshima 734-8551 Japan; 6 Department of Neurophysiology, Faculty of Medicine, University of Yamanashi, Yamanashi, 409-3898 Japan; 7 Department of Anatomy, Faculty of Medicine, Hokkaido University, Sapporo, Hokkaido 060-8638 Japan; 8 Laboratory for Animal Resources and Genetic Engineering, RIKEN Center for Developmental Biology, Kobe, Hyogo 650-0047 Japan; 9 International Research Center for Neurointelligence (WPI-IRCN), The University of Tokyo Institutes for Advanced Study (UTIAS), Bunkyo-ku, Tokyo 113-0033 Japan

**Keywords:** Classical conditioning, Molecular neuroscience

## Abstract

Classical eyeblink conditioning is a representative associative motor learning that requires both the cerebellar cortex and the deep cerebellar nucleus (DCN). Metabotropic glutamate receptor subtype 1 (mGluR1) is richly expressed in Purkinje cells (PCs) of the cerebellar cortex. Global mGluR1 knock-out (KO) mice show a significantly lower percentage of conditioned response (CR%) than wild-type mice in eyeblink conditioning, and the impaired CR% is restored by the introduction of mGluR1 in PCs. However, the specific roles of mGluR1 in major memory processes, including formation, storage and expression have not yet been defined. We thus examined the role of mGluR1 in these processes of eyeblink conditioning, using mGluR1 conditional KO (cKO) mice harboring a selective and reversible expression of mGluR1 in PCs. We have found that eyeblink memory is not latently formed in the absence of mGluR1 in adult mouse PCs. However, once acquired, eyeblink memory is expressed even after the depletion of mGluR1 in PCs. We thus conclude that mGluR1 in PCs is indispensable for the formation of eyeblink memory, while it is not required for the expression of CR.

## Introduction

Classical eyeblink conditioning provides a valuable paradigm for the study of associative motor learning and human neurological disorders^1–3^. Eyeblink conditioning involves the pairing of a conditioned stimulus (CS) with a blink-eliciting unconditioned stimulus (US). Naive animals initially exhibit unconditioned responses (URs) following the US. After repeated conditioning with a CS-US pair, they learn the association between the CS and the US and exhibit adaptively timed conditioned responses (CRs) before US onset. Eyeblink conditioning paradigms generally fall into two forms: delay, and trace^4^. In the delay paradigm, the CS and US temporally overlap and terminate simultaneously, while in the trace paradigm, a stimulus-free interval intervenes between the end of the CS and the onset of the US. Delay eyeblink conditioning critically depends on the intermediate cerebellum ipsilateral to the conditioned eye^5–7^, whereas trace eyeblink conditioning requires intact hippocampal function^8,9^. Many experiments of eyeblink conditioning using lesion^5,10,11^, pharmacological inactivation^12–15^, and mutant mice^16–19^ have shown that the cerebellum is important for the successful delay eyeblink conditioning. Nonetheless, there are still some debates about the brain structures where the eyeblink memory is formed and stored. Some groups suggest that the cerebellum is mostly involved in the proper expression of acquired CRs rather than formation or storage of eyeblink memory^12,20–22^.

**Figure 1:**
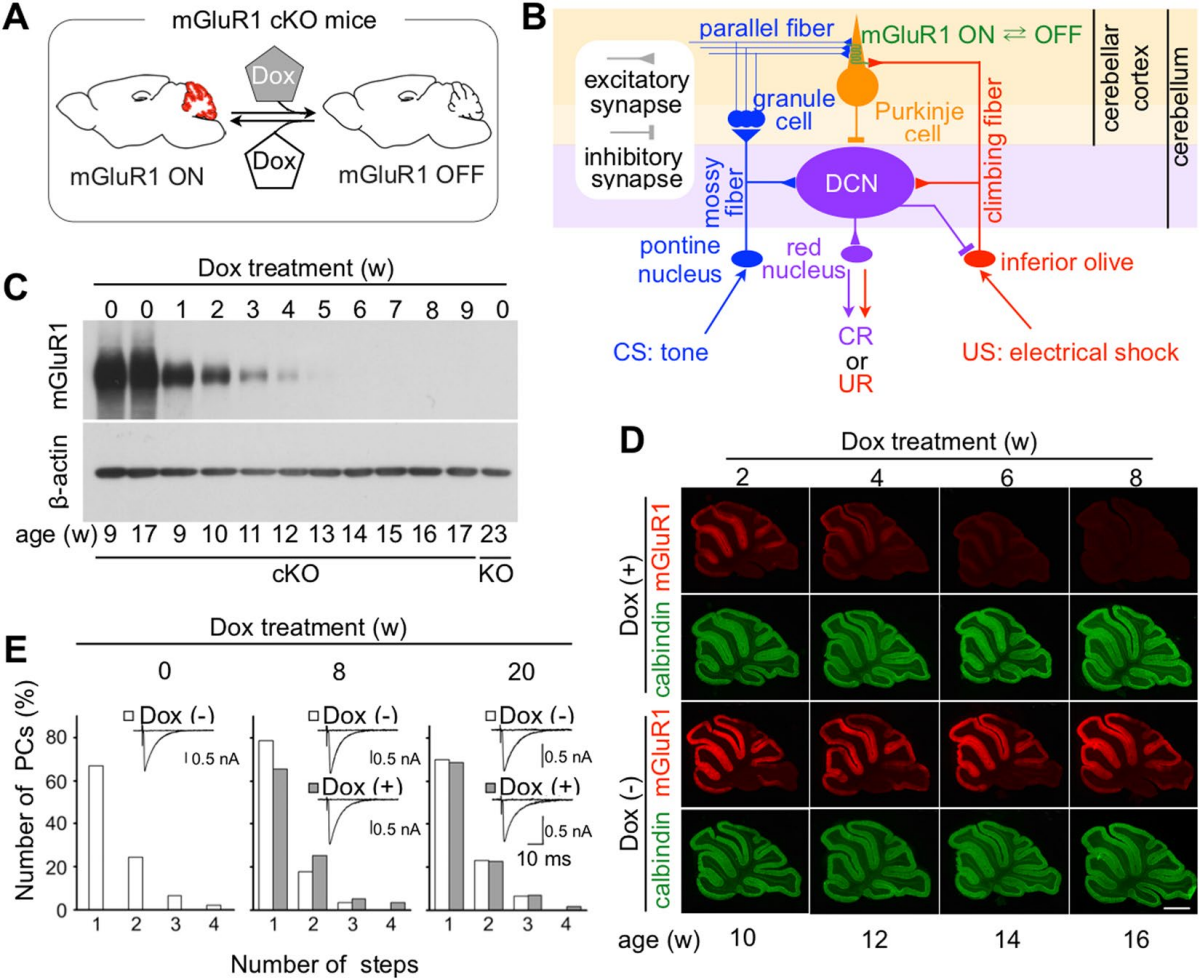
Climbing fiber innervation of PCs is normal after mGluR1 depletion in adult mGluR1 cKO mice. (**A**) mGluR1 in mGluR1 cKO PCs is reversibly inactivated by Dox administration. (**B**) Simplified schematic diagram of the circuits essential for eyeblink conditioning. Most interneurons have been omitted from this diagram. (**C**) Immunoblotting of cerebellar proteins from Dox-treated and untreated cKO mice with anti-mGluR1 and anti-β-actin antibodies. All lanes contain 10 µg of protein. The lane at the right end contains proteins from a global mGluR1 KO mouse^16^. (**D**) Immunostaining of cerebellar sagittal slices from Dox-treated and untreated cKO mice with anti-mGluR1 (red) and calbindin (green) antibodies. Scale bar: 1 mm. (**E**) Summary histograms showing the number of discrete steps for climbing fiber-mediated EPSCs (CF-EPSCs) recorded from mGluR1 cKO PCs before (left, 45 cells), 8 weeks after [middle, 56 cells for Dox (−) and 55 cells for Dox (+)], and 20 weeks after [right, 30 cells for Dox (−) and 57 cells for Dox (+)] Dox treatment. No significant difference was found between Dox-treated and untreated mice after 8 weeks (p = 0.110, Mann-Whitney U-test) or 20 weeks (p = 0. 839). Insets show representative traces of CF-EPSCs recorded from PCs.

Metabotropic glutamate receptor subtype 1 (mGluR1) is richly expressed in cerebellar Purkinje cells (PCs)^23,24^, which receive glutamatergic excitatory inputs from parallel fibers (PFs) and climbing fibers (CFs). Global mGluR1 knock-out (KO) mice show deficits in motor coordination, long-term depression (LTD) at PF-PC synapses, and developmental transition from multiple to monoinnervation of PCs by CFs^16,25–29^. Furthermore, global mGluR1 KO mice show a significantly lower percentage of CR (CR%) than wild-type mice in both delay- and trace- eyeblink conditioning paradigms^16,27^. Introduction of mGluR1 into cerebellar PCs restored the deficit in delay eyeblink conditioning in mGluR1 KO mice^27^. This clearly demonstrates that the mGluR1 within the cerebellar PCs is essential for CR acquisition in delay eyeblink conditioning. However, in these mouse models with irreversible gene-regulation, the specific roles of mGluR1 in the formation and expression of delay eyeblink memory have not been able to separately define. To address this issue, we utilized a genetic method that enables reversible KO of mGluR1 in a PC-specific manner^30^, in which mGluR1 was selectively and reversibly expressed in PCs (Fig. 1A).

## Results

### Depletion of mGluR1 protein in adulthood does not disrupt the neural circuits required for associative motor learning, but does alter Purkinje cell activities *in vivo*

**Figure 2:**
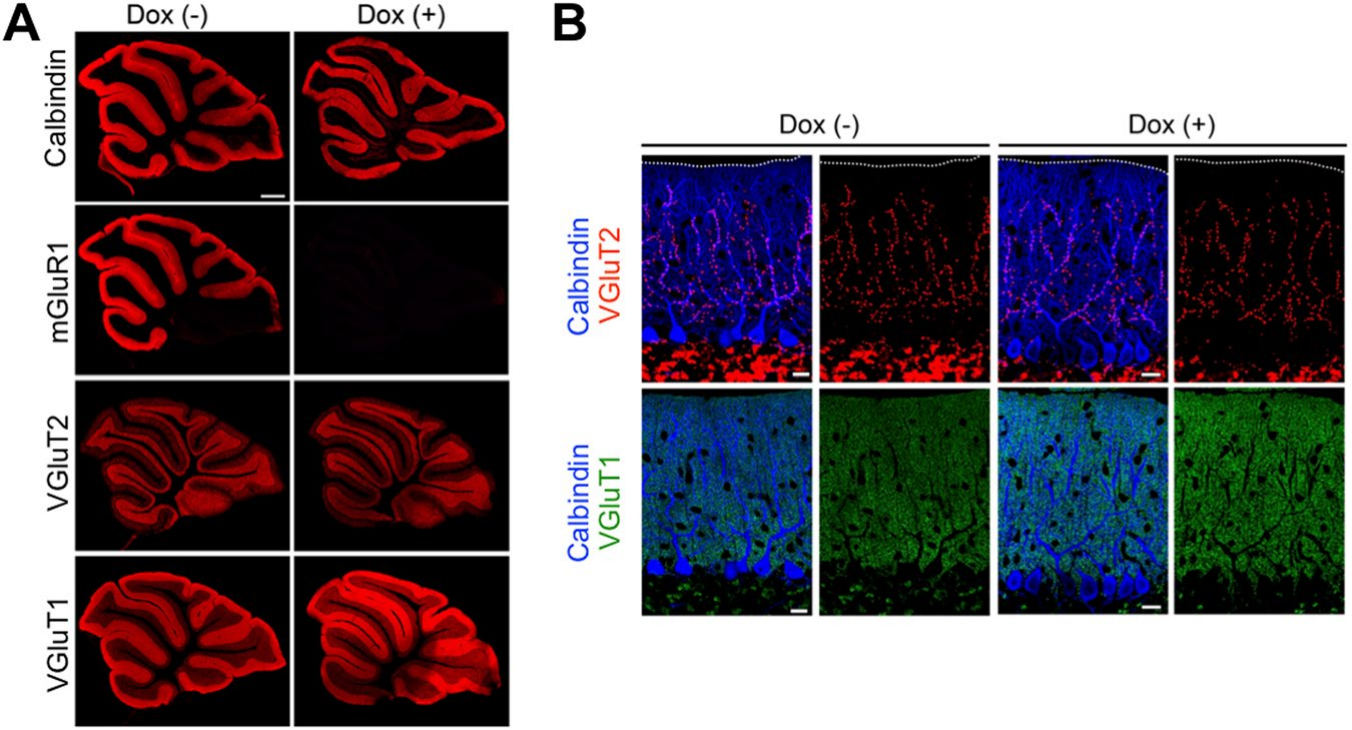
Normal distribution of parallel fiber and climbing fiber terminals 37 weeks after the initiation of Dox administration. (**A**) Immunofluorescence of cerebellar parasagittal sections from untreated and Dox-treated cKO mice 37 weeks after the beginning of Dox administration. Sections were stained with antibodies against mGluR1, calbindin, VGluT1 and VGluT2. In Dox-treated cKO mice, immunoreactivity for mGluR1 was abolished, whereas that for calbindin, VGluT1, or VGluT2 was comparable to untreated mGluR1 cKO mice. Scale bar: 0.5 mm. (**B**) Triple immunofluorescence for calbindin (blue), VGluT1 (green) and VGluT2 (red). There was no apparent difference between untreated and Dox-treated mGluR1 cKO mice in the distribution of parallel fiber or climbing fiber terminals. Scale bars: 20 µm.

The neural circuits required for the CR in delay eyeblink conditioning are well characterized^2,31,32^. The US pathway is composed of CF projections from inferior olivary neurons to PCs and CF collaterals to DCN neurons (Fig. 1B)^33^. In global mGluR1 KO mice, elimination of surplus CFs during early postnatal development is impaired, and multiple CF innervation persists into adulthood^25^. Therefore we first examined whether depletion of mGluR1 from adult PCs affects CF innervation of PCs, which may influence the neural circuits for eyeblink conditioning. Eight weeks after the beginning of doxycycline (Dox) administration, mGluR1 protein was completely depleted (Fig. 1C,D). No significant difference was found between Dox-treated and untreated cKO mice in the frequency distribution of PCs in terms of the number of discrete steps of CF-mediated excitatory postsynaptic current (EPSC) (Fig. 1E), indicating no change in CF innervation of PCs. Furthermore, immunohistochemical analyses confirmed that Dox-treated and untreated cKO mice had no apparent difference in the distribution of PF or CF terminals, even 37 weeks after the initiation of Dox treatment (Fig. 2). These results indicate that depletion of mGluR1 protein from PCs in adulthood does not disrupt the organization of CF-PC and PF-PC synapses.

We also examined the firing properties of PCs in cKO mice *in vivo*, and found that simple spike activity was preserved in cKO mice during Dox-induced depletion of mGluR1 from PCs, although the level was lower than that of control mice (Table 1). The decrease in simple spike activity in cKO mice was due to an increased ratio of PCs showing intermittent/rhythmic burst firing [81% (22/27 cells in 3 mice in cKO mice compared with 48% (13/27 cells in 3 mice) in control] (See Supplementary Fig. S1). By contrast, complex spike activity of PCs in cKO mice was higher than that in control mice (Table 1).

**Table 1:** Action potential firing of Purkinje cells in the mGluR1 cKO mice under the ketamine/xylazine anesthesia.

	Firing rate of simple spikes (Hz)	Firing rate of complex spikes (Hz)	Number of PCs (cells)
Dox (−)	19.8 ± 3.1	0.23 ± 0.07	27
Dox (+)	11.7 ± 1.9*	0.52 ± 0.04**	27

The mGluR1 cKO mice were treated with Dox between 8 and 16 weeks of age. Simple spike activity *in vivo* was preserved in mGluR1 cKO mice during Dox-induced depletion of mGluR1 from PCs, although the level was lower than that of control mice. Complex spike activity of mGluR1 cKO mice *in vivo* was higher than that of control mice. *P < 0.05, **P < 0.01.

### Associative motor memory is not latently formed without mGluR1 in PCs of adult mice

**Figure 3:**
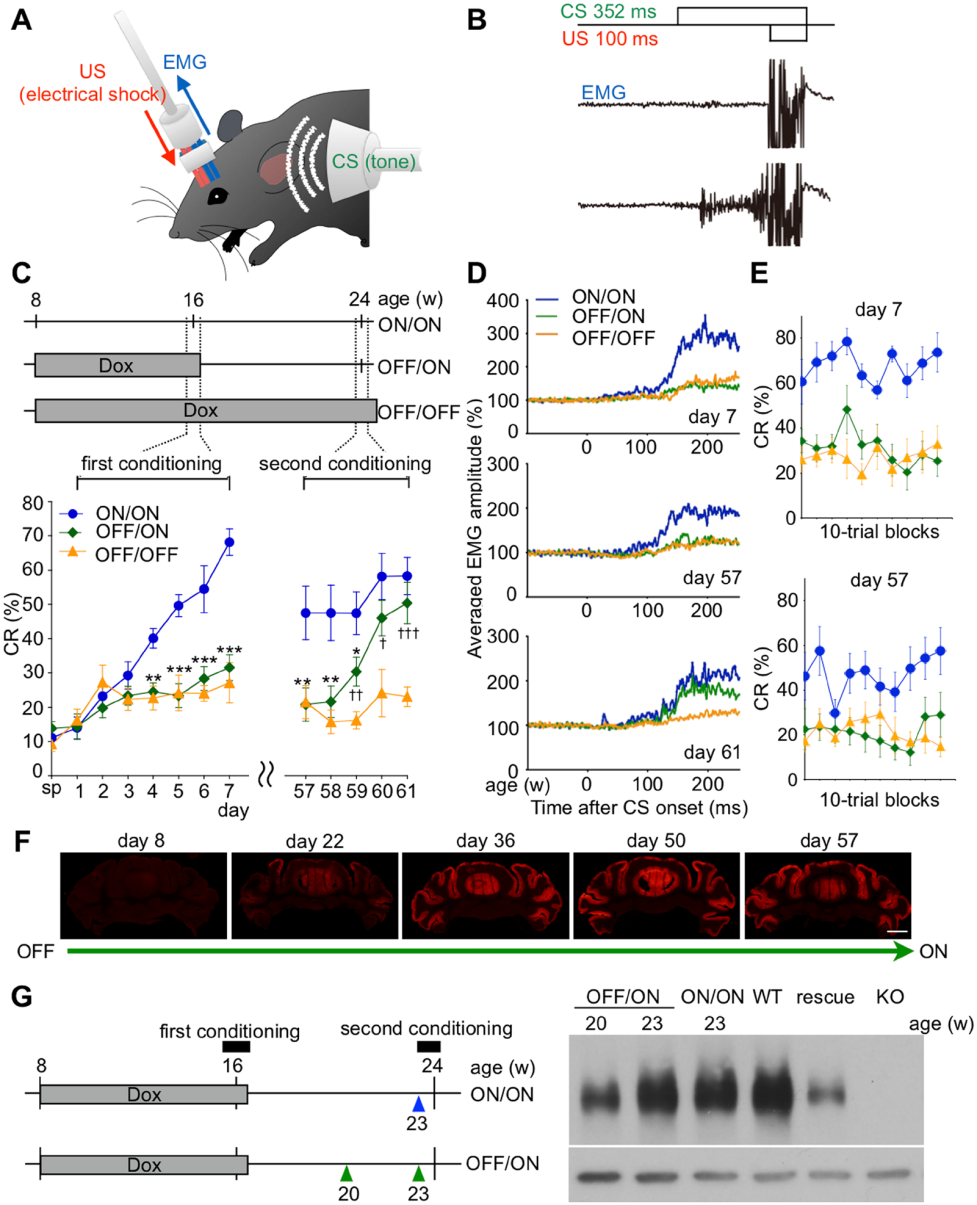
Associative motor memory is not formed latently without mGluR1 in PCs of adult mice. (**A**) Scheme of an animal implanted with electrodes for recording the electromyographic activity. (**B**) Typical raw EMG recordings of mGluR1 cKO mice were described under the CS-US representations. Upper EMG is an example of raw EMG before conditioning. Lower EMG is an example of raw EMG confirmed as CR after conditioning. (**C**) Development of CR% in mGluR1 ON/ON mice (*n* = 10), mGluR1 OFF/ON mice (*n* = 10), and mGluR1 OFF/OFF mice (*n* = 10). Top panel indicates experimental design for the timing and period of eyeblink conditioning in the three mouse gropes. Gray boxes indicate the timing of DOX administration. Mouse age is indicated above the schedules. In mGluR1 ON/ON mice, mGluR1 was continuously expressed. In mGluR1 OFF/ON mice, mGluR1 was not expressed in the first conditioning (days 1–7) but was restored in the second conditioning (days 57–61). In mGluR1 OFF/OFF mice, mGluR1 was not expressed during the entire experimental period. ^*^p < 0.05, ^**^p < 0.01, ^***^p < 0.001 (ON/ON vs. OFF/ON). ^†^p < 0.05, ^††^p < 0.01, ^†††^p < 0.001 (OFF/ON vs. OFF/OFF). (**D**) Averaged EMG amplitudes on days 7, 57, and 61. All EMG amplitudes obtained in one session (100 trials) were summed, representing the overall response pattern. (**E**) The intraday CR% for three groups of mGluR1 cKO mice on days 7 and 57. On day 57 the CR% for mGluR1 OFF/ON mice was as low as that for mGluR1 OFF/OFF mice in all blocks. (**F**) Expression of mGluR1 in mGluR1 OFF/ON mice. Coronal sections from mGluR1 cKO mice at different time points after Dox withdrawal were stained with antibody against mGluR1. (**G**) Immunoblotting of cerebellar proteins from mGluR1 cKO mice^30^ (OFF/ON and ON/ON) with anti-mGluR1 and anti-β-actin antibodies. Proteins from a wild-type mouse (WT), a transgenic mouse harboring L7-mGluR1a transgene (rescue)^26^ and a global mGluR1 KO mouse (KO)^16^ were also applied. The blots cropped from different parts of a same gel were separately shown with a white space. All lanes contain 10 µg of protein. Scale bar: 1 mm. Data are represented as mean ± SEM.

**Figure 4:**
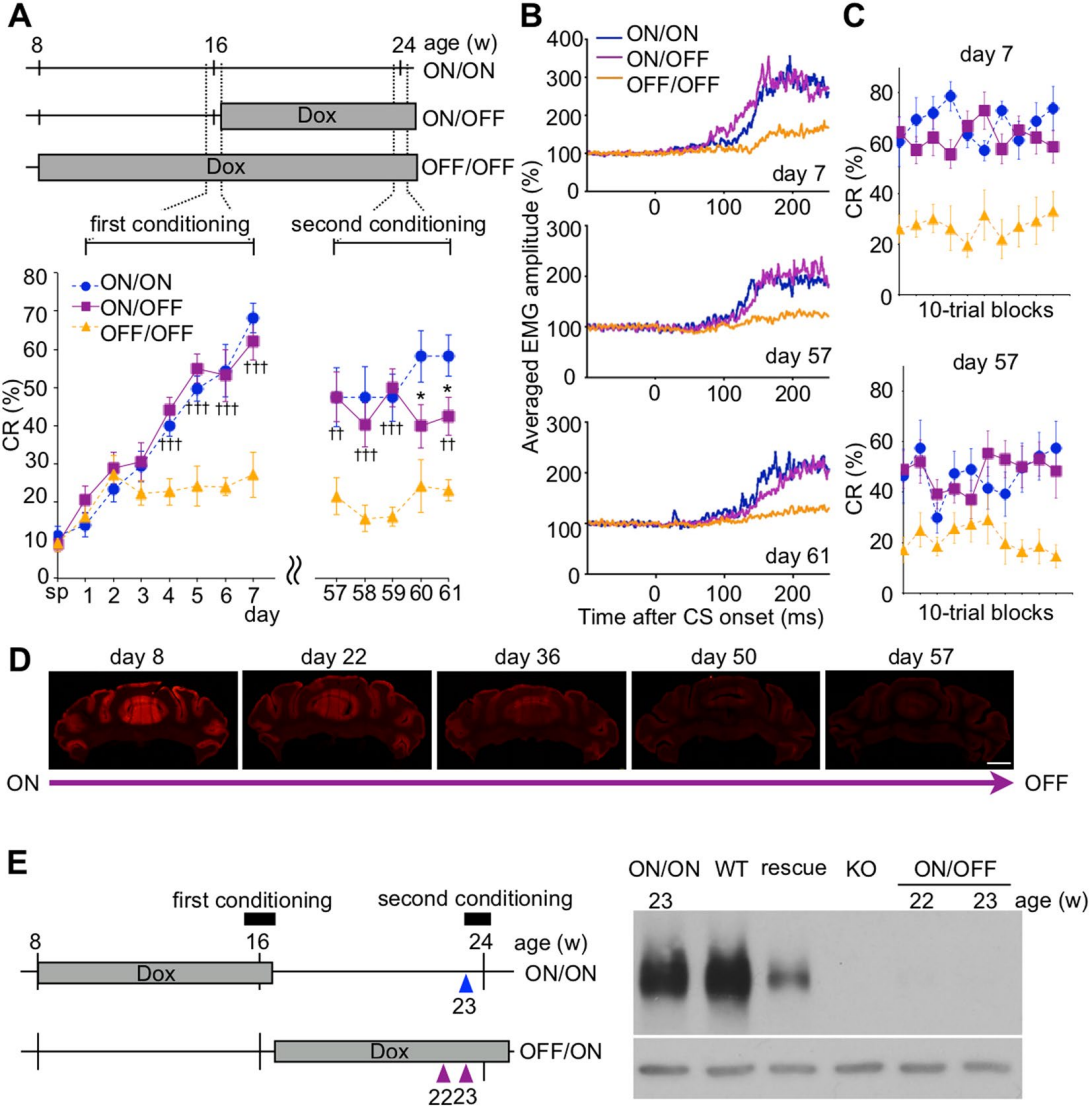
Acquired motor memory is retrieved without mGluR1 in PCs. (**A**) Development of CR% in mGluR1 ON/ON mice (n = 10), mGluR1 ON/OFF mice (n = 10), and mGluR1 OFF/OFF mice (n = 10). Top panel indicates experimental design for the timing and period of eyeblink conditioning in the three mouse gropes. Gray boxes indicate the timing and period of DOX administration. Mouse age is indicated above the schedules. In mGluR1 ON/OFF mice, mGluR1 was expressed without Dox in the first conditioning (days 1–7), but the expression was blocked with Dox in the second conditioning (days 57–61). The CR% for mGluR1 ON/OFF mice was as high as those for mGluR1 ON/ON mice on day 57, even though no mGluR1 protein remained in PCs, demonstrating that mGluR1 in PCs is dispensable for the expression of once acquired eyeblink memory. Controls were mGluR1 ON/ON and mGluR1 OFF/OFF mice, and the data for these mice are identical to those shown in Fig. 3. ^*^p < 0.05 (ON/ON vs. ON/OFF). ^††^p < 0.01, ^†††^p < 0.001 (ON/OFF vs. OFF/OFF). (**B**) Averaged EMG amplitudes on days 7, 57, and 61. All EMG amplitudes obtained in one session (100 trials) were summed, representing the overall response pattern. (**C**) The intraday CR% for mGluR1 ON/OFF mice on days 7 and 57. On day 57, the CR% for mGluR1 ON/OFF mice was as high as that for mGluR1 ON/ON mice in all blocks. Data are presented as in Fig. 3. (**D**) Expression of mGluR1 in mGluR1 ON/OFF mice. Coronal sections from mGluR1 cKO mice at different time points after the start of Dox administration were stained with antibody against mGluR1. (**E**) Immunoblotting of cerebellar proteins from mGluR1 cKO mice^30^ (ON/ON and ON/FF) with anti-mGluR1 and anti-β-actin antibodies. Proteins from a wild-type mouse (WT), a transgenic mouse harboring L7-mGluR1a transgene (rescue)^26^ and a global mGluR1 KO mouse (KO)^16^ are identical to those shown in Fig. 3G. The blots cropped from different parts of a same gel were separately shown with a white space. All lanes contain 10 µg of protein. Scale bar: 1 mm. Data are represented as mean ± SEM.

For mouse eyeblink conditioning analyses, we used four different groups of mGluR1 cKO mice in which the mGluR1 transgene was activated at different time points: (1) the transgene was activated throughout the first and second eyeblink conditioning (mGluR1 ON/ON); (2) the transgene was inactivated prior to the first conditioning and the inactivation continued to the end of the second conditioning (mGluR1 OFF/OFF); (3) the transgene was inactivated prior to the first conditioning, then activated prior to the second conditioning (mGluR1 OFF/ON); and (4) the transgene was activated until the end of the first conditioning and then inactivated prior to the second conditioning (mGluR1 ON/OFF) (Figs 3 and 4).

First we examined whether mGluR1 in PCs is required for the formation of eyeblink memory (Fig. 3A–E). The CR% for mGluR1 ON/ON mice increased up to 68.2 ± 3.9% during the first conditioning (between days 1 and 7). In contrast, the CR% for mGluR1 OFF/ON (31.6% ± 3.8% on day 7) and that for mGluR1 OFF/OFF (27.1% ± 5.9% on day 7) mice, both of which lacked mGluR1 in their PCs during the first conditioning, were significantly lower (Fig. 3C; repeated-measures ANOVA, Session × Group interaction, F(12, 162) = 8.40, p < 0.001, a main effect of session, F(2, 27) = 14.31, p < 0.001). Averaged eyelid electromyogram (EMG) amplitudes for these mice were also much lower than for mGluR1 ON/ON mice on the last day of the first conditioning (day 7, Fig. 3D). Importantly, on the first day of the second conditioning (day 57), the CR% and EMG amplitude for mGluR1 OFF/ON mice were as low as those for mGluR1 OFF/OFF mice (Fig. 3C,D). Indeed, on day 57, the CR% for mGluR1 OFF/ON mice was as low as that for mGluR1 OFF/OFF mice in all 10-trial blocks, demonstrating that eyeblink memory was not latently formed without mGluR1 (Fig. 3E: repeated-measures ANOVA, Session × Group interaction, F(18, 243) = 1.56, p = 0.072, a main effect of session, F(2, 27) = 6.05, p = 0.0067). Moreover, mGluR1 OFF/ON mice learned the CR as if they had been naive after the expression of mGluR1 was restored by withdrawal of Dox for 7 weeks (Fig. 3F). The CR% for mGluR1 OFF/ON mice on the last day of the second conditioning (day 61, 50.4% ± 6.1%) reached the same level as that for mGluR1 ON/ON mice on the fifth day of the first conditioning (day 5, 49.6% ± 3.2%). There was no significant difference between the learning curve for mGluR1 OFF/ON mice (between days 57 and 61) and that for mGluR1 ON/ON mice (between days 1 and 5) (Fig. 3C; repeated-measures ANOVA, Session × Group interaction, F(4, 72) = 0.41, p = 0.80, a main effect of session, F(1, 18) = 0.63, p = 0.44).

The impaired CR in mGluR1 OFF/ON mice suggest either that mGluR1 is required for formation of eyeblink memory or that mGluR1 is necessary for expression of the CR while it is dispensable for formation of eyeblink memory. If the latter is the case, the CR% for mGluR1 OFF/ON mice on the first day of the second conditioning (day 57) would be comparable to that for mGluR1 ON/ON mice on the same day. However, the CR% for mGluR1 OFF/ON mice on day 57 was as low as that for mGluR1 OFF/OFF mice. At one week before the second conditioning (23 weeks of age), the amount of mGluR1 protein of mGluR1 OFF/ON mouse is comparable to that of mGluR1 ON/ON mouse, whereas that of the rescued mouse was drastically reduced, reported in the previous report^27^ (Fig. 3G, See Supplementary Fig. S2). This result suggests that mGluR1 expression was adequately restored in mGluR1 OFF/ON mouse after 7 weeks from Dox withdrawal. These results exclude the possibility that mGluR1 in PCs is required only for the expression of CR, but rather indicate that mGluR1 is essential for the formation of associative motor memory.

### Acquired associative motor memory is expressed without mGluR1 in PCs

Next, we examined whether mGluR1 in PCs is required for expression of the acquired motor memory (Fig. 4A–C). In the first conditioning, the CR% increased progressively in mGluR1 ON/OFF mice as well as in mGluR1 ON/ON mice (Fig. 4A). In spite of the lack of mGluR1 in PCs on the first day of the second conditioning, the CR% for mGluR1 ON/OFF mice was as high as that for mGluR1 ON/ON mice. There was no significant difference between the learning curve for mGluR1 ON/OFF mice and that for mGluR1 ON/ON mice in the second conditioning (Session × Group interaction, F(4, 72) = 2.12, p = 0.087, a main effect of session, F(1, 18) = 1.14, p = 0.301). The EMG amplitudes (Fig. 4B) and CR% in all 10-trial blocks (Fig. 4C: Session × Group interaction, F(18, 243) = 2.00, p = 0.010, a main effect of session, F(2, 27) = 5.31, p = 0.011) for mGluR1 ON/OFF mice were also as high as those for mGluR1 ON/ON mice on the same day, even though no mGluR1 protein remained in PCs (day 57, Fig. 4D). In addition, at 2 weeks before the second conditioning (22 weeks of age), the amount of mGluR1 protein from mGluR1 ON/OFF mice is not detectable, indicating that mGluR1 expression is completely suppressed until on the first day of the second conditioning (Fig. 4E, See Supplementary Fig. S2). These results strongly suggest that mGluR1 in PCs is dispensable for the expression of once acquired eyeblink memory.

## Discussion

In the present study, we used mGluR1 cKO mice bearing inducible and reversible expression of mGluR1 specifically in PCs to identify the role of mGluR1 in PCs in the memory formation and expression, separately. We have clearly demonstrated that mGluR1 in PCs of the cerebellar cortex is indispensable for the formation of eyeblink memory. mGluR1 OFF/ON mice showed impaired CR due to the lack of mGluR1 during the first conditioning (Fig. 3C–E). A seemingly contradictory result has been previously reported using reversible neurotransmission blocking (RNB) of granule cells leading to suppression of synaptic transmission from PFs to PCs in mice^34^. Blockade of glutamate release from PFs induced severe impairment of CR acquisition; however, restoration of granule cell transmission resulted in a rapid increase in CR% from the beginning of the second conditioning in CR% after a 2–week interval. These results suggested that a latent memory of eyeblink conditioning was formed even without PF to PC transmission, and that normal PF to PC transmission was needed for expression of the stored memory of eyeblink conditioning. When granule cell to PC synaptic transmission is blocked, mGluR1 signalling in PCs would be blocked. We therefore expected to obtain results similar to those after RNB of granule cell to PC synaptic transmission from our analyses of the mGluR1 OFF/ON mice. However, our results are contrary to this expectation (Fig. 3C–E). One clear difference between the RNB and present studies is the level of simple spike activity of PCs. During blockade of granule cell transmission in the RNB mice, no simple spike activity was found in PCs^34^ and, therefore, inhibition of DCN neurons from PCs was almost absent. In marked contrast to the previous study, simple spike activity *in vivo* was preserved in our mGluR1 cKO mice during Dox-induced depletion of mGluR1 from PCs, although the level was lower than that of control mice (Table 1). In the RNB mice, DCN neurons were free from cortical inhibition during blockade of granule cell transmission, and therefore the latent memory might be ectopically formed in the DCN. However, because of the lack of cortical inhibition, basal activities of DCN neurons would be too high to faithfully increase their firing rates in response to the CR. Because of this “ceiling effect,” CR would not be expressed during conditioning, despite the formation of memory trace in the DCN. We also found that complex spike activity was elevated in cKO mice compared with that in control mice (Table 1). In cKO mice, most PCs showed intermittent/rhythmic burst firing (See Supplementary Fig. S1), which would result in rebound firing of DCN neurons^35,36^. This would lead to elevated complex spike activity, given the fact that activation of DCN neurons not only inhibits inferior olive neurons by direct nucleo-olivary projections but also excites them via a disynaptic excitatory pathway through the mesodiencephalic junction^37^.

Many previous studies on mutant mice with deficient LTD at PF-PC synapses in the cerebellar cortex including global mGluR1 KO mice^16,17,38–44^ and several reports on spontaneous mutant mice including PC degeneration (*pcd*) mice and Lurcher (*Lc*) mice^18,45^ have revealed that the cerebellar cortex is important for normal acquisition of conditioned eyeblink responses. However, it was unclear whether memory of eyeblink conditioning is temporally or persistently stored in the cerebellar cortex. We have also demonstrated that mGluR1 in PCs of the cortex is dispensable for the expression of once acquired eyeblink memory. The CR% for mGluR1 ON/OFF mice was normal on day 57 in the second conditioning without mGluR1 even though they also exhibited significantly lower CR% on days 60–61 (Fig. 4A). In adult mice, administration of Dox for 7 weeks was sufficient to induce ataxia^30^, suggesting that mGluR1 is functionally depleted in the cerebellum. The poor CR improvement of mGluR1 ON/OFF mice could be due to impaired re-formation of motor memory.

These results suggest that eyeblink memory is first encoded in the cerebellar cortex, but the memory trace is transferred to brain regions other than the cerebellar cortex. Our result is consistent with a recent optogenetic study showing that LTD in the cerebellar cortex is directly responsible for adaptation of horizontal optokinetic response (HOKR) and vestibulo-ocular reflex, other representative paradigms of cerebellum-dependent motor learning^46^. In the mouse HOKR, a functional memory trace of short-term adaptation is formed initially in the cerebellar cortex and subsequently transferred to the vestibular nuclei to be stored as persistent memory through *de novo* protein synthesis^47,48^. In the eyeblink conditioning, the CS and US signals are conveyed to both PCs in the cerebellar cortex and neurons in the DCN, and the convergence of these two signals is capable of inducing persistent plastic changes in the responsiveness of both neuron types^49–51^. The DCN is a candidate site where eyeblink memory is stored as persistent memory. In contrast to the RNB mice, DCN neurons were tonically suppressed by cortical inhibition and presumably maintained normal basal activities during blockade of mGluR1 expression in our mGluR1 ON/OFF mice. The CR was therefore expressed faithfully based on the memory trace that had been stored in the DCN before the second conditioning.

In conclusion, the present study demonstrates that mGluR1 is essential for the formation but not expression of associative eyeblink memory and that the delay eyeblink memory is formed in the cerebellar cortex in an mGluR1-dependent manner. This result will give a clear answer to the long-standing controversy regarding whether the cerebellum is the site for formation of delay eyeblink memory.

## Materials and methods

### Animals

All animal experiments and methods were approved by and performed in accordance with the relevant guidelines and regulations of the Institutional Animal Care and Use Committee of Kobe University Graduate School of Medicine (P041207R), the Institutional Animal Care and Use Committee of RIKEN Kobe Campus (AH13-03-7 and AH13-03-17), the animal investigation committee at Tokushima Bunri University (P-26), and the animal welfare committees of The University of Tokyo (P13–25). Generation of global mGluR1 KO (mGluR1^−/−^)^16^ mice bearing L7-tTA (a tetracycline-controlled transactivator) and TRE (a tetracycline response element) -mGluR1a transgenes (mGluR1^−/−^/L7-tTA/TRE-mGluR1a: mGluR1 cKO mice; C57BL/6 N background) was described previously^30^. Animals were housed in a room with controlled humidity, temperature, and a 12:12 h light-dark cycle. All mice were given ad libitum access to food and water. Mice of either sex were used for all experiments. Age-matched mice were allocated into experimental groups randomly.

### Administration of doxycycline

The mGluR1 cKO mice were treated with 200 µg/ml of Dox (doxycycline hyclate; Sigma, St Louis, MO) in drinking water between 8 and 16 weeks of age and/or between 16 and 24 weeks of age. The water was delivered in dark bottles to protect Dox from light and changed twice a week. At 16 and 24 weeks of age, mGluR1 cKO mice underwent eyeblink conditioning.

### Immunohistochemical analysis

After perfusion with 4% paraformaldehyde in 0.1 M phosphate buffer (pH 7.4), cerebella were cut on a microslicer (VT 1000S-V1.0E; Leica Microsystems, Nussloch, Germany) into 50 µm-thick parasagittal sections. Alternately, cerebella were immersed in 30% sucrose solution overnight and cut on a freezing microtome (FX-801, COPER, Kanagawa, Japan) into 50 µm–thick parasagittal or coronal sections. Free-floating sections were incubated with primary antibodies overnight, followed by incubation with fluorescent secondary antibodies for 2 h. Images were taken with a fluorescence microscope (BZ-8000; Keyence, Osaka, Japan for Figs 1D, 3F and 4D) or confocal laser-scanning microscope (FV1000; Olympus, Tokyo, Japan for Fig. 2). A guinea pig anti-mGluR1a antibody (1 µg/ml)^52^ and a rabbit anti-calbindin antibody (Chemicon International, Temecula; used at a dilution of 1: 500) were used for Figs 1D, 3F and 4D. A guinea pig anti-mGluR1a antibody (1 µg/ml)^52^, a goat anti-calbindin antibody (1 µg/ml), a rabbit anti-vesicular glutamate transporter 1 (VGluT1) antibody (1 μg/ml), and a guinea pig anti-VGluT2 antibody (0.5 μg/ml) were used for Fig. 2. The above primary antibodies were described in previous reports, unless otherwise noted^52–54^. For immunofluorescence, Cy3-, Cy5-, Alexa488-labeled species-specific secondary antibodies were used at a dilution of 1:200 (Jackson Immunoresearch, West Grove, PA; Invitrogen).

### Immunoblot analysis

Cerebella were homogenized in a buffer containing 0.32 M sucrose, 10 mM Tris-HCl (pH 7.4), 1 mM EDTA, and protease inhibitors (Complete Mini, EDTA-free; Roche, Mannheim, Germany). Ten micrograms of proteins were separated by SDS-PAGE and transferred to Immobilon-P membrane (Millipore, Billerica, MA). The membrane was probed with mouse anti-mGluR1a antibody (BD Biosciences, San Jose, CA) and an anti-β-actin antibody (Sigma, St Louis, MO) followed by an anti-mouse HRP-conjugated secondary antibody. Bound antibodies were visualized with ECL Plus detection reagents (GE Healthcare Bio-Sciences Corp, Piscataway, NJ).

### Electrophysiological recording

Parasagittal cerebellar slices (250-µm thick) were prepared from Dox-treated and untreated mGluR1 cKO mice. Slices were perfused at 31 °C with standard saline containing (in mM): NaCl, 125; KCl, 2.5; CaCl_2_, 2; MgSO_4_, 1; NaH_2_PO_4_, 1.25; NaHCO_3_, 26; glucose, 20; and bicuculline methochloride, 0.01 (Tocris Cookson, Bristol UK). This standard bathing solution was bubbled with 95% O_2_ and 5% CO_2_. Whole-cell recordings were made from visually identified PCs in lobules I-VIII using an upright microscope (Olympus BX50WI or Zeiss Axioskop). Detailed procedures are described in previous reports^25,55^.

### *In vivo* Two-photon imaging guided cell-attached loose-patch recording

Dox treatment was conducted for the mGluR1 cKO mice between 8 and 16 weeks of age. Surgery and recording were performed according to the procedures described previously^56^. Recording from PCs of cerebellar simple lobule was conducted in ketamine and xylazine anesthetized animals. PCs were visualized with ‘negative contrast’ by perfusing the extracellular space with fluorescent dye, which was included in the internal solution of the patch pipette. The same electrode was subsequently used for cell-attached loose-patch recording. The signal was recorded, amplified, low-pass-filtered at 10 kHz, high-pass-filtered at 100 Hz. The sampling rate for analysis was 50 kHz.

### Surgery for eyeblink conditioning

All mGluR1 cKO mice underwent surgery at 15 weeks of age. Surgery was performed as described previously^44^. Mice were anesthetized with ketamine (80 mg/kg, i.p. Sankyo, Tokyo, Japan) and xylazine (20 mg/kg, i.p. Bayer, Tokyo, Japan). Four Teflon-coated stainless-steel wires (100 µm in diameter, A-M Systems, WA, USA) were implanted subcutaneously under the left eyelid. Two of the wires were used to deliver the US and the remaining two to record an electromyogram (EMG) from the musculus orbicularis oculi, which is responsible for eyelid closure.

### Eyeblink conditioning

All mGluR1 cKO mice were trained in delay eyeblink conditioning, in which the CS overlaps and coterminates with the US, for 7 days as the first conditioning, followed by 5 days as the second conditioning. A tone of 352 ms duration (1 kHz, 80 dB) was used as CS and electrical shock with 100 ms duration (100 Hz square pulses) as US (interstimulus interval = 252 ms). The US intensity was carefully determined as the minimal current amplitude required to elicit an eyeblink response with constant amplitude and was adjusted daily for each animal (less than 0.5 mA). Experiments were conducted during the light phase of LD cycle in a container (10 cm in diameter) placed in a sound- and light-attenuating chamber. Two days were allotted for recovery and another 2 days for acclimation of the animals to the conditioning chamber (50 min with no stimulus) after the surgery (see Supplemental Experimental Procedure). Daily training consisted of 100 trials grouped in 10 blocks. Conditioning sessions consisted of 10 CS-only (every 10th trial) and 90 CS-US paired trials. The spontaneous eyeblink frequency was measured during 100 ‘no stimulus’ trials in the second session (day 0) for acclimation before the conditioning experiment began. CR amplitude was calculated as the amplitude at the time 50 ms before the US. Data were analyzed as described previously^57^.

### Statistical analysis

An appropriate sample size was computed when the study was being designed. All data are presented as mean ± SEM. Data were statistically analyzed using Mann-Whitney U test, or t-test for electrophysiological analysis. Data were analyzed by repeated-measure ANOVA for eyeblink conditioning, and a post-hoc Tukey-Kramer or Bonferroni test was used when significant differences were found. The difference was considered significant when the *p* value was less than 0.05. The statistical Package for the GraphPad Prism 6 (GraphPad Software Inc., La Jolla, CA, USA) was used to analyze the data for the behavioral tests.

## Supplementary information

Supplementary Information
