# Supplementary material for: mGluR1 in cerebellar Purkinje cells is essential for the formation but not expression of associative eyeblink memory
Source: Sci Rep. 2019 May 14;9:7353. doi: 10.1038/s41598-019-43744-z (PMC6517439; doi:10.1038/s41598-019-43744-z)
Supplement: Supplementary file 1 — Supplementary Information [file 41598_2019_43744_MOESM1_ESM.docx]

Supplementary Information

**mGluR1 in cerebellar Purkinje cells is essential for the formation but not expression of associative eyeblink memory**

Harumi Nakao^1,2, #^, Yasushi Kishimoto^3, #,*^, Kouichi Hashimoto^4,5^, Kazuo Kitamura^4,6^, Miwako Yamasaki^7^, Kazuki Nakao^2,8^, Masahiko Watanabe^7^, Masanobu Kano^4,9^, Yutaka Kirino^3^, and Atsu Aiba^1,2,*^

^1^Division of Molecular Genetics, Department of Physiology and Cell Biology, Kobe, University Graduate School of Medicine, Kobe, Hyogo 650-0017, Japan. ^2^Laboratory of Animal Resources, Center for Disease Biology and Integrative Medicine, Graduate School of Medicine, The University of Tokyo, Bunkyo-ku, Tokyo 113-0033, Japan. ^3^Laboratory of Neurobiophysics, Kagawa School of Pharmaceutical Sciences, Tokushima Bunri University, Sanuki, Kagawa 769-2193, Japan. ^4^Department of Neurophysiology, Graduate School of Medicine, The University of Tokyo, Bunkyo-ku, Tokyo 113-0033, Japan. ^5^Department of Neurophysiology, Graduate School of Biomedical & Health Sciences, Hiroshima University, Minami-ku, Hiroshima 734-8551, Japan. ^6^Department of Neurophysiology, Faculty of Medicine, University of Yamanashi, Yamanashi 409-3898, Japan. ^7^Department of Anatomy, Hokkaido University School of Medicine, Sapporo, Hokkaido 060-8638, Japan. ^8^Laboratory for Animal Resources and Genetic Engineering, RIKEN Center for Developmental Biology, Kobe, Hyogo 650-0047, Japan

^9^International Research Center for Neurointelligence (WPI-IRCN), The University of Tokyo Institutes for Advanced Study (UTIAS), Bunkyo-ku, Tokyo 113-0033, Japan. **^#^**These authors contributed equally to this work.


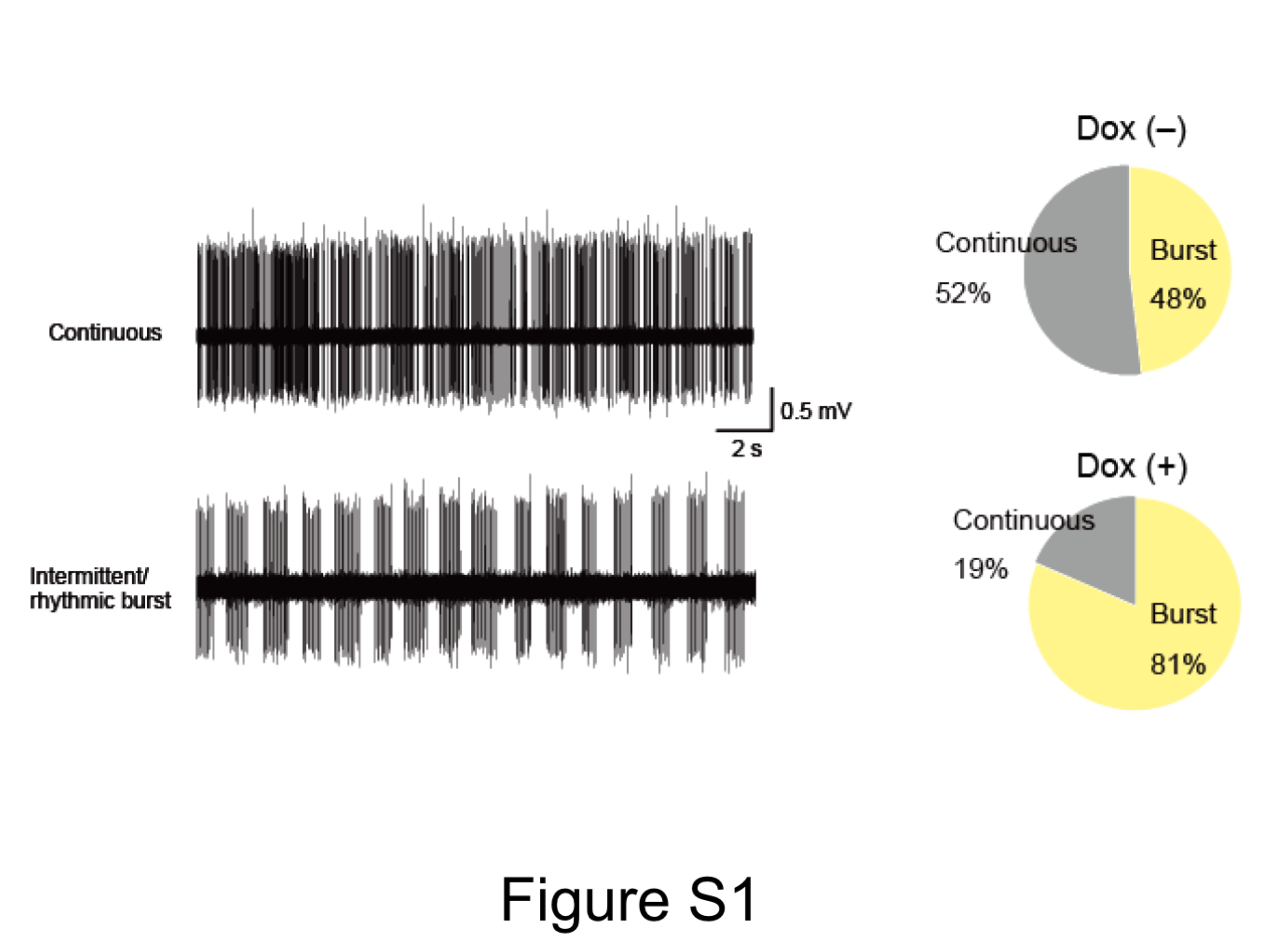


**Supplementary Figure S1. Firing activity of Purkinje cells in the mGluR cKO mice in vivo.**

Left, representative spiking activities of Purkinje cells showing continuous (upper) and intermittent/rhythmic burst (lower) firing. Right, ratio of Purkinje cells showing continuous and burst firing in control [Dox(–)] and cKO [Dox(+)] mice.

**
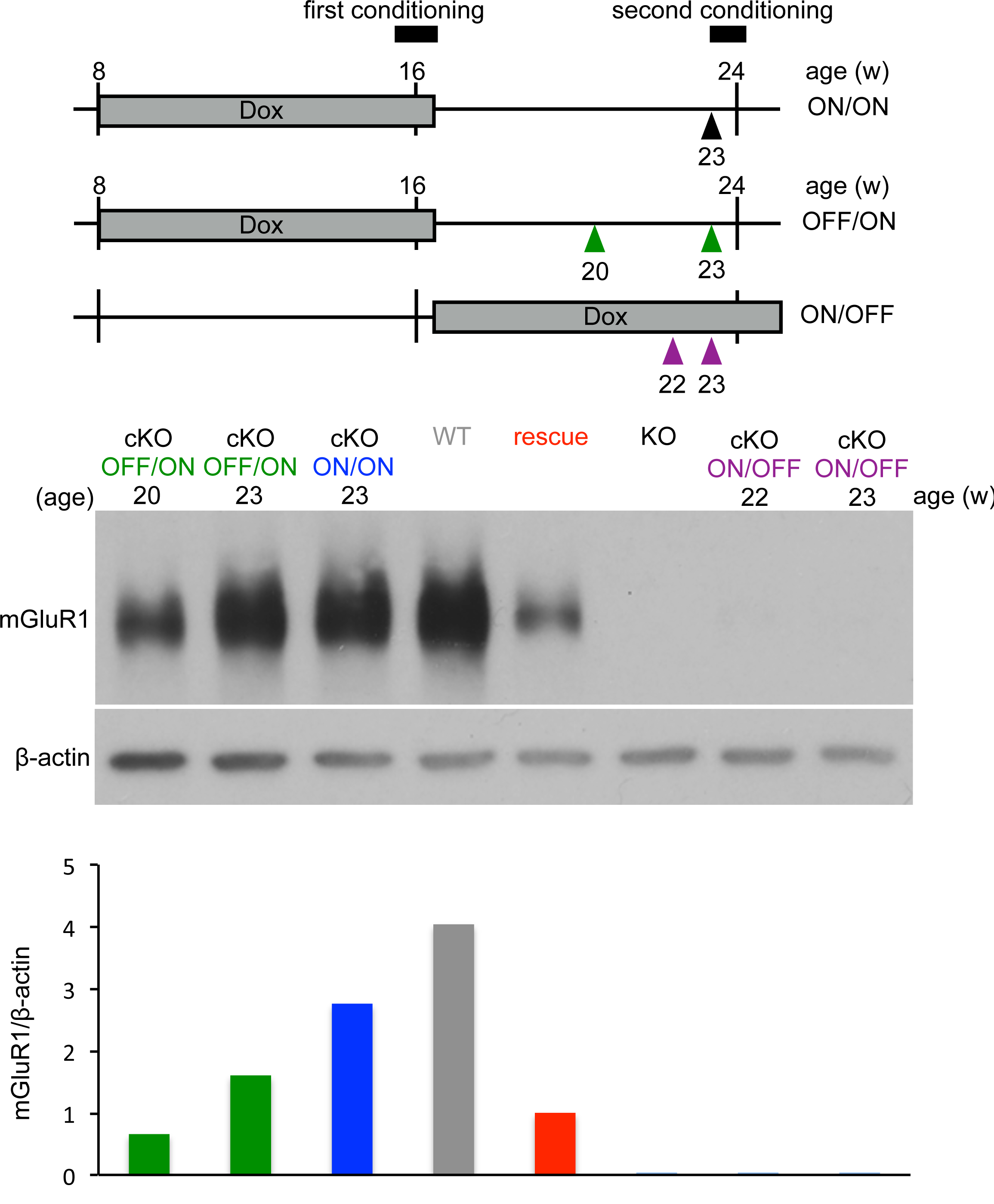
**

**Supplementary Figure S2. Restoration or depletion of mGluR1 protein in mGluR1 OFF/ON and ON/OFF mice.**

Immunoblotting of cerebellar proteins from mGluR1 cKO mice[^1^](#_ENREF_1) (OFF/ON, ON/ON and ON/OFF), a wild-type mouse (WT), a transgenic mouse harboring L7-mGluR1a transgene (rescue)[^2^](#_ENREF_2) and a global mGluR1 KO mouse (KO)[^3^](#_ENREF_3) with anti-mGluR1 and anti-β-actin antibodies are shown in Figs. 3G and 4E. The relative ratio of mGluR1/β-actin was calculated and shown as the fold of control (The ratio of mGluR1/β-actin in “rescue” was defined as 1).

**Supplemental experimental procedures**

***Immunoblot analysis***

The blots (Figs. 3G and 4E) were quantified using the Image J 1.48v software (Wayne Rasband, National Institute of Health, USA).

**Supplemental references**

1 Nakao, H., Nakao, K., Kano, M. & Aiba, A. Metabotropic glutamate receptor subtype-1 is essential for motor coordination in the adult cerebellum. *Neurosci. Res.* **57**, 538-543, doi:10.1016/j.neures.2006.12.014 (2007).

2 Ichise, T. *et al.* mGluR1 in cerebellar Purkinje cells essential for long-term depression, synapse elimination, and motor coordination. *Science* **288**, 1832-1835 (2000).

3 Aiba, A. *et al.* Deficient cerebellar long-term depression and impaired motor learning in mGluR1 mutant mice. *Cell* **79**, 377-388 (1994).
